# Bacterial Lysis through Interference with Peptidoglycan Synthesis Increases Biofilm Formation by Nontypeable *Haemophilus influenzae*

**DOI:** 10.1128/mSphere.00329-16

**Published:** 2017-01-18

**Authors:** Sara Marti, Carmen Puig, Alexandra Merlos, Miguel Viñas, Marien I. de Jonge, Josefina Liñares, Carmen Ardanuy, Jeroen D. Langereis

**Affiliations:** a Microbiology Department, Hospital Universitari Bellvitge, IDIBELL-University of Barcelona, Barcelona, Spain; b Laboratory of Pediatric Infectious Diseases, Radboud Center for Infectious Diseases, Radboud University Medical Center, Nijmegen, The Netherlands; c Research Network for Respiratory Diseases (CIBERES), ISCIII, Madrid, Spain; d Department of Pathology & Experimental Therapeutics, IDIBELL-University of Barcelona, L’Hospitalet de Llobregat, Spain; Escola Paulista de Medicina/Universidade Federal de São Paulo

**Keywords:** DNA, *Haemophilus influenzae*, biofilms, otitis media, peptidoglycan, postantibiotic effect

## Abstract

Nontypeable *Haemophilus influenzae* (NTHi) is an opportunistic pathogen that mainly causes otitis media in children and community-acquired pneumonia or exacerbations of chronic obstructive pulmonary disease in adults. A large variety of studies suggest that biofilm formation by NTHi may be an important step in the pathogenesis of this bacterium. However, the underlying mechanisms involved in this process are poorly elucidated. In this study, we used a transposon mutant library to identify bacterial genes involved in biofilm formation. The growth and biofilm formation of 4,172 transposon mutants were determined, and the involvement of the identified genes in biofilm formation was validated in *in vitro* experiments. Here, we present experimental data showing that increased bacterial lysis, through interference with peptidoglycan synthesis, results in elevated levels of extracellular DNA, which increased biofilm formation. Interestingly, similar results were obtained with subinhibitory concentrations of β-lactam antibiotics, known to interfere with peptidoglycan synthesis, but such an effect does not appear with other classes of antibiotics. These results indicate that treatment with β-lactam antibiotics, especially for β-lactam-resistant NTHi isolates, might increase resistance to antibiotics by increasing biofilm formation.

**IMPORTANCE** Most, if not all, bacteria form a biofilm, a multicellular structure that protects them from antimicrobial actions of the host immune system and affords resistance to antibiotics. The latter is especially disturbing with the increase in multiresistant bacterial clones worldwide. Bacterial biofilm formation is a multistep process that starts with surface adhesion, after which attached bacteria divide and give rise to biomass. The actual steps required for *Haemophilus influenzae* biofilm formation are largely not known. We show that interference with peptidoglycan biosynthesis increases biofilm formation because of the release of bacterial genomic DNA. Subinhibitory concentrations of β-lactam antibiotics, which are often prescribed to treat *H. influenzae* infections, increase biofilm formation through a similar mechanism. Therefore, when β-lactam antibiotics do not reach their MIC *in vivo*, they might not only drive selection for β-lactam-resistant clones but also increase biofilm formation and resistance to other antimicrobial compounds.

## Abstract (precis)

Most, if not all, bacteria form a biofilm, a multicellular structure that protects them from antimicrobial actions of the host immune system and affords resistance to antibiotics. The latter is especially disturbing with the increase in multiresistant bacterial clones worldwide. Bacterial biofilm formation is a multistep process that starts with surface adhesion, after which attached bacteria divide and give rise to biomass. The actual steps required for *Haemophilus influenzae* biofilm formation are largely not known. We show that interference with peptidoglycan biosynthesis increases biofilm formation because of the release of bacterial genomic DNA. Subinhibitory concentrations of β-lactam antibiotics, which are often prescribed to treat *H. influenzae* infections, increase biofilm formation through a similar mechanism. Therefore, when β-lactam antibiotics do not reach their MIC *in vivo*, they might not only drive selection for β-lactam-resistant clones but also increase biofilm formation and resistance to other antimicrobial compounds.

## INTRODUCTION

*Haemophilus influenzae* is a Gram-negative human-restricted bacterium that forms part of our oropharyngeal microbiota, where it resides without causing disease symptoms (1). *H. influenzae* strains are categorized into six distinct serotypes (a through f) based on the polysaccharide capsule. Unencapsulated or nontypeable *H. influenzae* (NTHi) strains are also isolated from patients. NTHi is most frequently associated with inflammatory diseases of the human mucosa, including otitis media (OM), sinusitis, and exacerbations of chronic obstructive pulmonary disease (COPD) (2–4).

Many bacterial pathogens form biofilms during infection of the human host. The ability of NTHi to form biofilms *in vivo* during disease was first visualized on tympanostomy tubes collected from children with OM (5). Since then, biofilm growth of NTHi has been observed in the middle ear mucosa of children with OM (6), as well as in bronchoalveolar lavage fluid from individuals with cystic fibrosis (7) and bronchiectasis (8). This growth state protects bacteria against efficient killing by the immune system (9, 10), as well as enhances resistance to antimicrobials (11, 12).

Many studies have attempted to establish a link between biofilm formation by NTHi and its ability to cause disease. For instance, we found that especially NTHi strains collected from patients with OM have the ability to form biofilms *in vitro* (13). This is consistent with *in vivo* experiments, where biofilms in the ears of chinchillas were associated with bacterial persistence (14). Although there are indications that biofilm formation is related to pathogenesis, the molecular mechanisms underlying NTHi biofilm formation are still not completely understood (15). It has been shown that biofilms that are formed both *in vitro* and *in vivo* contain significant amounts of DNA, either bacterial or host derived (14, 16). It has been shown that the DNA-associated protein DNABII plays a relevant role in stabilizing the DNA matrix within the NTHi biofilm and that antisera against DNABII rapidly disrupt biofilms (17).

To identify novel bacterial genes that are associated with NTHi biofilm formation *in vitro*, we performed an unbiased genome-wide mutagenesis screening by using a clinical NTHi isolate. We provide evidence that disruption of peptidoglycan synthesis and recycling, either by making gene deletion mutants or by using subinhibitory concentrations of β-lactam antibiotics, increases sensitivity to bacterial lysis, releasing bacterial DNA that contributes to biofilm formation *in vitro*.

## RESULTS

### Transposon mutant library biofilm screening.

We generated transposon mutants of NTHi strain 13/4, a biofilm-forming isolate from an oropharyngeal swab collected from a healthy child (18). This strain was selected because it formed a biofilm *in vitro*, it had low incorporation of phosphorylcholine into the lipooligosaccharide (19), and transformation with mutagenized DNA obtained a sufficient number of transposon mutants.

We determined *in vitro* biofilm formation and growth in two experiments with 1,806 and 2,366 colonies, and the data were combined for a total of 4,172 colonies. Eight hundred ten transposon mutants (310 and 500 in the first and second experiments, respectively) showed normal growth (optical density at 620 nm [OD_620_] <35% lower than that of the wild type [WT]) but altered biofilm formation (*A*_560_ at least 1.5-fold different from that of the WT) and were screened for a second round. Thirty-three out of 53 mutants (29 and 24 in the first and second experiments, respectively) showed consistent significant differences in biofilm formation after the transformation of genomic DNA into the NTHi 13/4 WT strain.

The transposon insertion site was successfully identified for 30 transposon mutants (Table 1). For the transposon mutants with at least a 2-fold decrease in biofilm formation (R2866 gene loci R2866_1635 [*mltC*] and R2866_1770 [*lppB*]) or 2-fold increased biofilm formation (R2866 gene loci R2866_0135 [*ponA*], R2866_0223 [*ampG*], R2866_0638 [*amiB*], R2866_0673 [*mrdA*], R2866_1548 [*dsbA*], and R2866_1640), directed gene deletion mutants of NTHi strain R2866, whose genome has been sequenced, were constructed.

**TABLE 1:** Transposon mutants with increased or decreased biofilm formation

R2866 gene locus	Gene name	Product	No. of mutants	% of WT biofilm formation
R2866_1548	*dsbA*	Thiol:disulfide interchange protein	3	332
R2866_0223	*ampG*	Peptidoglycan permease AmpG	3	307
R2866_0673	*mrdA*	Penicillin-binding protein 2	5	293
R2866_0135	*ponA*	Penicillin-binding protein 1A	3	279
R2866_0638	*amiB*	*N*-Acetylmuramoyl-l-alanine amidase	1	230
R2866_1640		Hypothetical protein	1	222
R2866_1798	*xerC*	Site-specific tyrosine recombinase	1	196
R2866_0262	*xerD*	Site-specific, tyrosine recombinase	1	179
R2866_1089	*hprA*	Glycerate dehydrogenase	1	176
R2866_0781	*moaC*	Molybdenum cofactor biosynthesis	1	166
R2866_1057	*potC*	Spermidine/putrescine ABC transporter, permease protein	1	163
R2866_0799	*lppC*	Lipoprotein C	1	142
R2866_1209	*gcvA*	Probable transcription activator	1	62
R2866_1826	*rep*	ATP-dependent DNA helicase	1	62
R2866_0451	*dnaQ*	DNA polymerase III, epsilon subunit	1	55
R2866_0833	*maeB*	NADP-dependent malic enzyme	1	51
R2866_1635	*mltC*	Membrane-bound lytic murein transglycosylase C	1	44
R2866_1770	*lppB*	Outer membrane antigenic lipoprotein B	3	7

The growth of the R2866Δ*amiB*, R2866Δ*mrdA*, R2866Δ*ampG*, R2866Δ*mltC*, and R2866Δ*lppB* biofilm mutants was significantly affected compared to that of WT R2866, although the differences were small (see Fig. S1A in the supplemental material). The deletions in R2866Δ*ampG*, R2866Δ*amiB*, and R2866Δ*mrdA* increased biofilm formation, whereas the deletions in R2866Δ*mltC* and R2866Δ*lppB* resulted in decreased biofilm formation (see Fig. S1B). The R2866Δ*mltC* and R2866Δ*lppB* mutants were able to form biofilm mass, but the biofilm was dislodged during washing, indicating that the adhesion of these mutants was affected.

10.1128/mSphere.00329-16.1 FIG S1  Stationary-phase cultures and biofilm formation determined for WT R2866 and gene deletion mutants. WT R2866 and gene deletion mutant overnight growth was determined by measuring OD_620_ (A), and biofilm formation was determined by measuring crystal violet light *A*_560_ (B). One-way analysis of variance with Dunnett’s multiple-comparison *post hoc* test was used for statistical analysis (NS, not significant; *, *P* < 0.05; **, *P* < 0.01; ***, *P* < 0.001). Download FIG S1, TIF file, 0.5 MB. Copyright © 2017 Marti et al. 2017 Marti et al. This content is distributed under the terms of the Creative Commons Attribution 4.0 International license.

Phase-contrast microscopic analysis of bacterial morphology showed that the R2866Δ*amiB* and R2866Δ*mrdA* mutants have an altered shape compared to that of WT R2866. The R2866Δ*mltC* mutant was longer and the R2866Δ*lppB* mutant formed very long chains compared to WT R2866, suggesting a defect in cell division (see Fig. S2).

10.1128/mSphere.00329-16.2 FIG S2  Phase-contrast microscopy of WT R2866 and gene deletion mutants. WT R2866 and gene deletion mutants were grown and visualized by phase-contrast microscopy. Download FIG S2, TIF file, 2.8 MB. Copyright © 2017 Marti et al. 2017 Marti et al. This content is distributed under the terms of the Creative Commons Attribution 4.0 International license.

Interestingly, mutations in R2866Δ*ponA*, R2866Δ*ampG*, R2866Δ*amiB*, and R2866Δ*mrdA* were involved in peptidoglycan synthesis and recycling for Gram-negative bacteria (20). Therefore, we further focused on these mutants, with R2866Δ*ampG* as a model because it showed the most prominent increase in biofilm formation.

### R2866Δ*ampG* mutant biofilm formation, adhesion, and aggregation.

**FIG 1:**
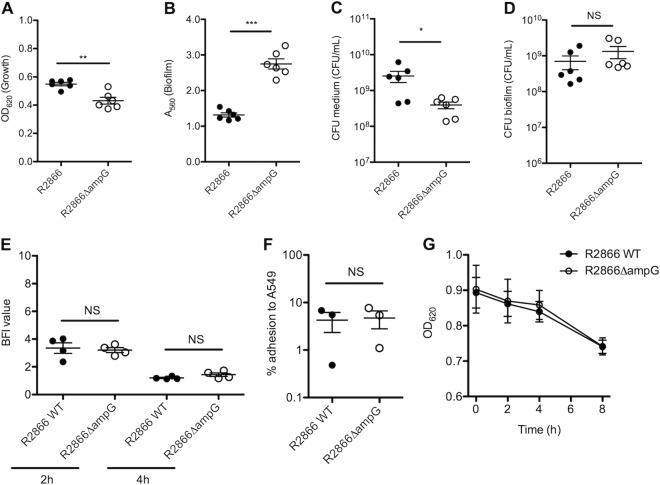
Growth, adhesion, biofilm formation, and aggregation of WT R2866 and the R2866Δ*ampG* gene deletion mutant. (A) Overnight growth determined by measuring OD_620_. (B) Biofilm formation determined by measuring crystal violet light *A*_560_. (C, D) Numbers of WT R2866 and R2866Δ*ampG* gene deletion mutant CFU in medium (C) and biofilm (D). (E) Initial adhesion to a solid surface determined by the biofilm ring test after 2 and 4 h of static growth at 37°C. The BFI was adjusted by the test software and is inversely proportional to the number of adherent bacteria. (F) Percent adhesion of WT R2866 and R2866Δ*ampG* gene deletion mutant bacteria to A549 lung epithelial cells after 2 to 3 h of incubation. (G) Aggregation of WT R2866 and the R2866Δ*ampG* gene deletion mutant in 5 ml of sBHI medium as determined by OD_620_ after 0, 2, 4, and 8 h of static incubation. An unpaired Student *t* test (A to F) or two-way analysis of variance with the Bonferroni *post hoc* test (G) was used for statistical analysis (NS, not significant; *, *P* < 0.05; **, *P* < 0.01; ***, *P* < 0.001).

The growth of the R2866Δ*ampG* mutant, determined by measuring the OD_620_ of an overnight culture, was significantly decreased compared to that of WT R2866 (Fig. 1A). In contrast, biofilm formation, as determined by measuring crystal violet biofilm staining, was increased more than 2-fold (Fig. 1B). The slight decrease in growth was likely due to a lower number of live bacteria because CFU counts in the medium were significantly lower (Fig. 1C), but this was not the case for CFU counts in the biofilm (Fig. 1D). This indicated that even though biofilm formation was increased, no increased bacterial CFU count was detected in the biofilm; however, we cannot exclude the formation of aggregates of multiple bacteria.

Adhesion is an essential first step in biofilm formation. The speed of initial adhesion to a solid surface was determined by the biofilm ring test after 2 and 4 h of static growth at 37°C. Both the R2866Δ*ampG* mutant and WT R2866 showed no differences in the level of initial adhesion *in vitro* (Fig. 1E) or in adhesion to respiratory epithelial cell line A549 (Fig. 1F). Bacterial aggregation can contribute to biofilm formation, but this was not significantly different between the R2866Δ*ampG* mutant and WT R2866 (Fig. 1G). Altogether, initial adhesion and aggregation were similar for the R2866Δ*ampG* mutant and WT R2866, whereas biofilm formation and the presence of genomic DNA in the biofilm were increased.

### Visualization of R2866Δ*ampG* mutant biofilm.

**FIG 2:**
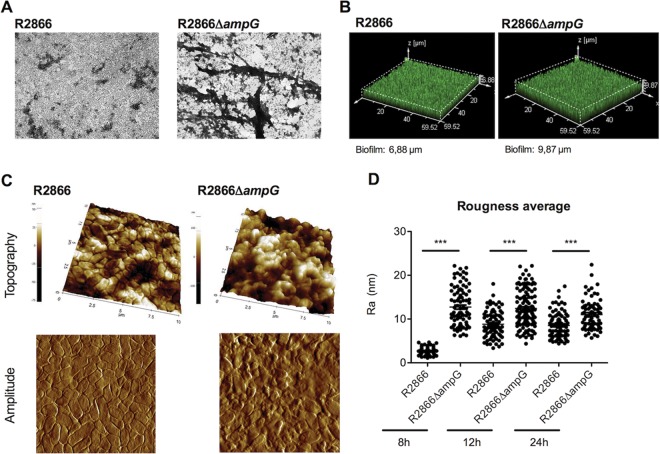
Microscopy analysis of WT R2866 and R2866Δ*ampG* gene deletion mutant biofilms. (A) WT R2866 and R2866Δ*ampG* gene deletion mutant biofilms stained with crystal violet and analyzed by light microscopy. (B) WT R2866 and R2866Δ*ampG* gene deletion mutant biofilms stained with SYTO 17 and analyzed by confocal microscopy. (C) Topography and error signals of WT R2866 and R2866Δ*ampG* gene deletion mutant biofilms determined by AFM. (D) Average roughness of WT R2866 and R2866Δ*ampG* in the biofilms calculated from topography images. An unpaired Student *t* test was used for statistical analysis (***, *P* < 0.001).

The biofilms formed by the WT R2866 and R2866Δ*ampG* mutant strains were visualized after 18 h of growth by crystal violet staining to determine whether any morphological differences between the two biofilms were present. Whereas single bacteria were often visible in the WT biofilm, the biofilm structure formed by the R2866Δ*ampG* was more complex, with large dense areas of crystal violet staining (Fig. 2A).

Confocal microscopy analysis of WT R2866 and the R2866Δ*ampG* mutant revealed that the biofilm of the R2866Δ*ampG* mutant was thicker, at almost 10 μm, than the WT R2866 biofilm, which was 7 μm thick (Fig. 2B).

Atomic force microscopy (AFM) visualization confirmed that WT R2866 differed strongly from the R2866Δ*ampG* mutant in different features such as cell size, cell shape, and the ability to produce biofilm, as shown in Fig. 2C and Table 2. Topography images are influenced by the bond order between the probe and the sample; in fact, it reflects the shape of the surface. The error signal imaging is partially based on extra deflection of the cantilever. Combination of the two images provides information about surfaces and their properties. Moreover, roughness determination, a measurement of cell integrity, revealed differences in roughness between WT R2866 and the R2866Δ*ampG* mutant at 8 h, whereas they tended to converge at longer periods of incubation, probably because of bacterial aging (Fig. 2D). After 12 and 24 h, differences in height, length, and amplitude cannot be determined since the bacteria are embedded in large amounts of extracellular matrix (Table 2).

**TABLE 2:** Cell size analysis by AFM

Time (h)	WT R2866				R2866Δ*ampG*			
	Ra (nm)	Ht (nm)	Amplitude (nm)	Length (nm)	Ra (nm)	Ht (nm)	Amplitude (nm)	Length (nm)
8	2.63 ± 0.95	29.13 ± 7.33	670.2 ± 60.3	1,221.1 ± 181.0	12.47 ± 5.05	181.8 ± 86.7	733.2 ± 87.8	1,030.2 ± 208.4
12	8.25 ± 3.14	ND^a^	ND	ND	11.36 ± 4.59	ND	ND	ND
24	8.17 ± 3.36	ND	ND	ND	10.92 ± 4.35	ND	ND	ND

a ND, not determined.

### Bacterial DNA contributes to increased biofilm formation by the R2866Δ*ampG* mutant.

**FIG 3:**
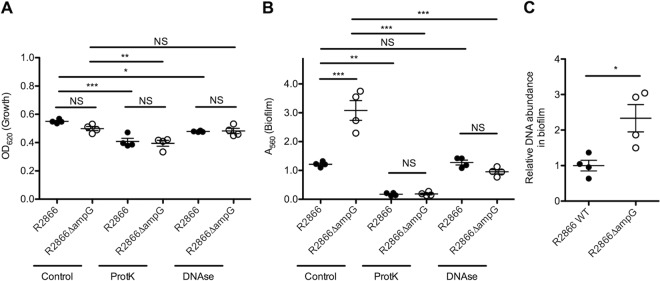
DNase and proteinase K treatment of WT R2866 and R2866Δ*ampG* gene deletion mutant biofilms. (A) Overnight growth determined by measuring OD_620_. (B) Biofilm formation by WT R2866 and the R2866Δ*ampG* gene deletion mutant determined by measuring crystal violet light *A*_560_ in the presence of proteinase K (100 μg/ml) or DNase (100 μg/ml). (C) Relative DNA abundance in the R2866Δ*ampG* gene deletion mutant compared to that in WT R2866 determined by qPCR detection of the *Haemophilus* protein D gene (*hpd*). One-way analysis of variance with Tukey’s multiple-comparison test was used for statistical analysis (NS, not significant; *, *P* < 0.05; **, *P* < 0.01; ***, *P* < 0.001).

Biofilm formation requires initial bacterial adhesion, which is mainly dependent on bacterial proteins and the presence of DNA, either bacterial or from the host (15). The contributions of protein-mediated adhesion and DNA to biofilm formation were determined by adding proteinase K or DNase to the growth medium, respectively. Adding proteinase K or DNase to the growth medium decreased bacterial growth as measured by determining the OD_620_ of an overnight culture, but no differences between WT R2866 and the R2866Δ*ampG* mutant were observed (Fig. 3A). Proteinase K treatment abrogated WT R2866 and R2866Δ*ampG* mutant biofilm formation completely (Fig. 3B), indicating that protein-mediated adhesion is required. DNase treatment decreased R2866Δ*ampG* mutant biofilm growth to the level of WT R2866, indicating that increased biofilm formation by the R2866Δ*ampG* mutant was dependent on extracellular genomic DNA (Fig. 3B).

Next, we measured the genomic DNA copy number in the biofilm by quantitative PCR (qPCR) and found a significantly greater DNA abundance in the R2866Δ*ampG* mutant biofilm than in the WT R2866 biofilm (Fig. 3C).

### The R2866Δ*ampG* mutant is more sensitive to bacterial lysis during static growth.

The static growth of WT R2866 and that of the R2866Δ*ampG* mutant were similar up to 5 h, but at later time points, the growth of the R2866Δ*ampG* mutant stayed behind (see Fig. S3A), which was also observed in CFU counts (see Fig. S3B). When fold increases in OD_620_ and CFU counts were calculated, the growth of WT R2866 was the same by both measurements (see Fig. S3C), whereas the CFU counts of the R2866Δ*ampG* mutant decreased compared to its OD_620_ (see Fig. S3D). These data might indicate that there was more bacterial lysis of the R2866Δ*ampG* mutant, contributing to an increase in OD_620_ but not in viable bacterial cell counts.

10.1128/mSphere.00329-16.3 FIG S3  Growth of WT R2866 and the R2866Δ*ampG* gene deletion mutant determined by OD measurement and CFU counting. The growth of WT R2866 and the R2866Δ*ampG* gene deletion mutant was determined by OD_620_ measurement (A) and CFU counting (B). (C, D) Fold increases in OD_620_ and CFU counts of WT R2866 (C) and the R2866Δ*ampG* mutant (D). Two-way analysis of variance and the Bonferroni *post hoc* test were used for statistical analysis (*, *P* < 0.05; **, *P* < 0.01; ***, *P* < 0.001). Download FIG S3, TIF file, 0.5 MB. Copyright © 2017 Marti et al. 2017 Marti et al. This content is distributed under the terms of the Creative Commons Attribution 4.0 International license.

**FIG 4:**
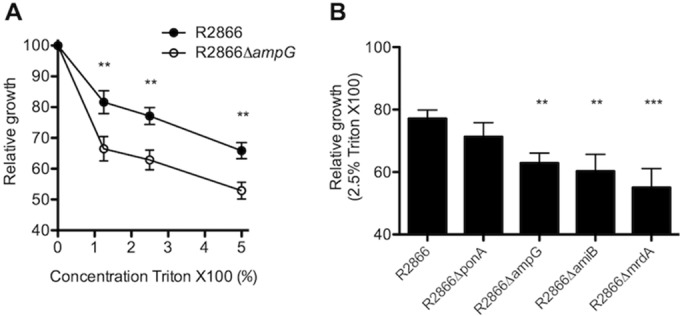
Relative growth of WT R2866 and the R2866Δ*ampG* gene deletion mutant with Triton X-100. (A, B) Relative growth of WT R2866 and the R2866Δ*ampG* gene deletion mutant (A) and other gene deletion mutants (B) in sBHI with 1, 2.5, and 5% Triton X-100 compared to that in sBHI. Two-way analysis of variance and the Bonferroni *post hoc* test (A) and one-way analysis of variance with Dunnett’s multiple-comparison *post hoc* test (B) were used for statistical analyses (NS, not significant; *, *P* < 0.05; **, *P* < 0.01; ***, *P* < 0.001).

To measure sensitivity to bacterial lysis, we cultured WT R2866 and the R2866Δ*ampG* mutant with Triton X-100, which weakens the membrane. The growth of WT R2866 was decreased by 1 to 5% Triton X-100; however, this difference was even greater in the R2866Δ*ampG* mutant (Fig. 4A). Increased sensitivity to Triton X-100 was also observed in other peptidoglycan synthesis-related mutants (R2866Δ*amiB*, R2866Δ*mrdA*), indicating that tampering with peptidoglycan biosynthesis increased sensitivity to Triton X-100 (Fig. 4B).

### β-Lactam antibiotic interference with peptidoglycan synthesis increases WT R2866 biofilm formation.

**FIG 5:**
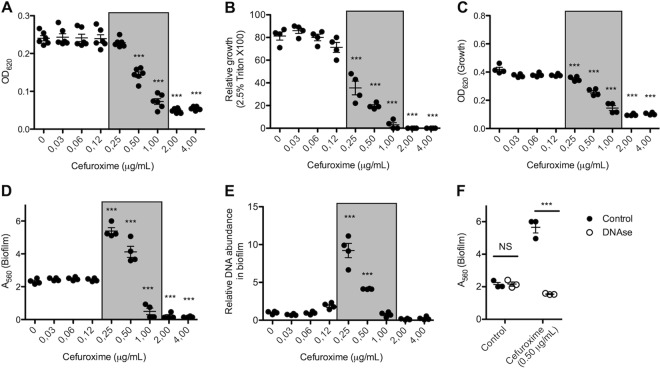
β-Lactam antibiotics increase bacterial lysis, relative DNA content, and biofilm formation. (A) Growth of WT R2866 with cefuroxime was determined after 3 h. (B) Relative growth of WT R2866 after 3 h with cefuroxime in sBHI with 2.5% Triton X-100 compared to that in sBHI. (C, D) Overnight growth with cefuroxime determined by measuring OD_620_ (C) and level of biofilm formation with cefuroxime determined by measuring crystal violet light *A*_560_ (D). (E) Relative DNA abundance in biofilm determined by qPCR detection of the *Haemophilus* protein D gene (*hpd*). (F) Biofilm formation by WT R2866 with and without 0.5 mg/ml cefuroxime with or without 100 μg/ml DNase determined by measuring crystal violet light *A*_560_. One-way analysis of variance with Dunnett’s multiple-comparison *post hoc* test (A to E) and two-way analysis of variance with the Bonferroni *post hoc* test (F) were used for statistical analyses (NS, not significant; *, *P* < 0.05; **, *P* < 0.01; ***, *P* < 0.001).

Previously, it was shown that subinhibitory concentrations of β-lactam antibiotics increased biofilm formation by NTHi (11). Since β-lactam antibiotics, like the gene deletions in our R2866Δ*ampG*, R2866Δ*amiB*, and R2866Δ*mrdA* mutants, also interfere with peptidoglycan synthesis, we determined the effect of cefuroxime on the bacterial growth, Triton X-100 sensitivity, and biofilm formation of WT R2866. The growth of WT R2866 was significantly inhibited by 0.5 μg/ml cefuroxime and completely abrogated by 2 μg/ml cefuroxime (Fig. 5A). Interestingly, sensitivity to Triton X-100 was significantly increased by 0.25 to 1 μg/ml cefuroxime, indicating that these bacteria are more sensitive to bacterial lysis (Fig. 5B). But even more importantly, although overnight growth was decreased (Fig. 5C), biofilm formation significantly increased in the presence of 0.25 to 0.5 μg/ml cefuroxime (Fig. 5D). Similar results were obtained with other β-lactam antibiotics (amoxicillin-clavulanic acid, ampicillin, and penicillin G) but not with other classes of antibiotics (see Fig. S4). In accordance with the data on the R2866Δ*ampG* mutant, inhibition of peptidoglycan biosynthesis with 0.25 to 0.5 μg/ml cefuroxime increased the relative DNA abundance in the biofilm almost 10-fold (Fig. 5E) and DNase decreased biofilm formation to control levels (Fig. 5F), indicating that extracellular DNA is required for increased biofilm formation.

10.1128/mSphere.00329-16.4 FIG S4  Effects of antibiotics on bacterial growth and biofilm formation. The overnight growth of WT R2866 with various classes of antibiotics was determined by measuring OD_620_, and the level of biofilm formation was determined by measuring crystal violet light *A*_560_. Download FIG S4, TIF file, 0.8 MB. Copyright © 2017 Marti et al. 2017 Marti et al. This content is distributed under the terms of the Creative Commons Attribution 4.0 International license.

**FIG 6:**
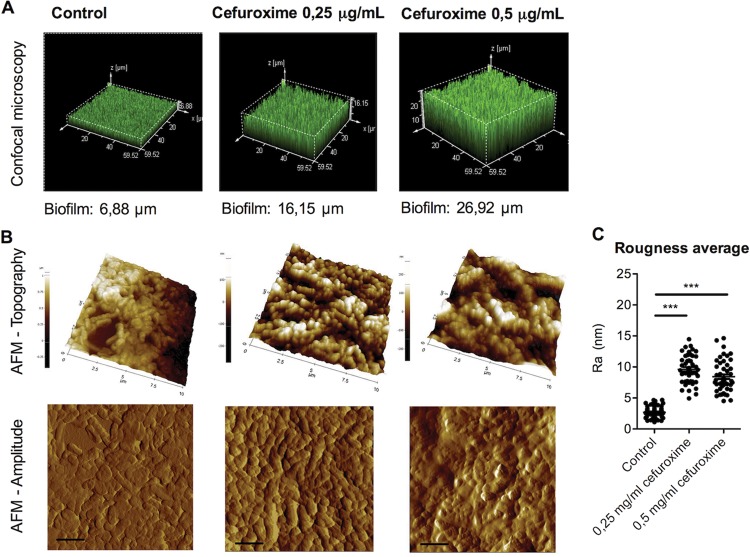
Microscopy analysis of WT R2866 biofilm grown with β-lactam antibiotics. (A) WT R2866 was grown overnight with or without 0.25 and 0.5 μg/ml cefuroxime, stained with SYTO 17, and analyzed by confocal microscopy. (B) Topography and error signals determined by AFM. (C) Average roughness of WT R2866 grown with or without 0.25 and 0.5 μg/ml cefuroxime in the biofilm calculated from topography images. One-way analysis of variance with Dunnett’s multiple-comparison *post hoc* test was used for statistical analysis (***, *P* < 0.001).

Next, we determined the effect of subinhibitory concentrations of the β-lactam antibiotic cefuroxime on bacterial growth and biofilm formation in 16 clinical NTHi isolates. Increased biofilm formation by 13 of 16 clinical isolates tested with cefuroxime was found (see Fig. S5), indicating that this is a broad phenotype. Consistent with the confocal microscopy analysis of the R2866Δ*ampG* mutant biofilm, cefuroxime increased the biofilm thickness of NTHi strain R2866 (Fig. 6A). At these sublethal concentrations of cefuroxime antibiotics, WT R2866 bacteria exhibited behavior quite similar to that of R2866Δ*ampG* mutants in biofilm-forming activity, as determined by AFM after 8 h (Fig. 6B and C).

10.1128/mSphere.00329-16.5 FIG S5  Effects of β-lactam antibiotics on the bacterial growth and biofilm formation of 16 clinical NTHi isolates. Overnight growth was determined by measuring OD_620_, and the level of biofilm formation was determined by measuring crystal violet light *A*_560_ for 16 clinical NTHi isolates (8 from sputum samples collected from COPD patients, 4 from middle ear fluid of children with OM, and 4 from the oropharynges of healthy children). Download FIG S5, TIF file, 1.3 MB. Copyright © 2017 Marti et al. 2017 Marti et al. This content is distributed under the terms of the Creative Commons Attribution 4.0 International license.

## DISCUSSION

Because the mechanisms that contribute to biofilm formation by NTHi are not completely understood (15), we aimed to identify genes required for NTHi biofilm formation. In this study, we screened >4,000 transposon mutants and evaluated their growth and biofilm formation in an *in vitro* static biofilm assay. Among the genes identified, *mltC* and *lppB* showed a significantly lower level of biofilm formation, possibly because of their elongated morphology. Both the MltC and LppB proteins contain lysostaphin-like metalloproteinase (LytM) domains, which are known to be involved in the cell division process (21). The R2866Δ*mltC* and R2866Δ*lppB* mutants revealed that these proteins play a crucial role in bacterial cell division, since both presented an aberrant cell morphology. These results are corroborated by a recently published study wherein deletion of *mltC* (*envC*) or *lppB* (*nlpD*) from NTHi strain 176 affected cell morphology (22). Decreased biofilm formation by R2866Δ*mltC* and R2866Δ*lppB* seemed to be due to decreased adhesion because the biofilm mass formed after overnight growth was dislodged easily during the washing steps in our biofilm staining procedure.

We identified multiple transposon mutants that showed greater biofilm formation than WT R2866. Among those genes, four (R2866Δ*ponA*, R2866Δ*ampG*, R2866Δ*amiB*, and R2866Δ*mrdA*) were related to peptidoglycan synthesis or recycling. We showed that interference with peptidoglycan synthesis or recycling by making directed gene deletion mutants increased bacterial lysis in the biofilm, supplying genomic DNA and thereby increasing biofilm mass. Interestingly, β-lactam antibiotics also act by blocking peptidoglycan synthesis; therefore, we tested the hypothesis that β-lactam antibiotics would also increase bacterial lysis and biofilm formation. Indeed, similar to the findings obtained with the directed gene mutants, β-lactam antibiotics increased sensitivity to Triton X-100 and biofilm formation at subinhibitory concentrations. This was also observed by Wu and coworkers, who showed that subinhibitory concentrations of β-lactam antibiotics increased biofilm formation by a variety of NTHi strains, which may be due to the upregulation of genes involved in glycogen production (11). We provide evidence that subinhibitory concentrations of β-lactam antibiotics increase bacterial lysis, thereby releasing bacterial genomic DNA, which contributes to biofilm formation *in vitro*. Subinhibitory concentrations of β-lactam antibiotics increased biofilm formation for 13 of 16 clinical NTHi isolates tested (see Fig. S5). The exact reasons why biofilm formation by three clinical NTHi isolates was not increased is not known. Two isolates showed a low biofilm formation level under the control condition; therefore, it is possible that initial attachment was not sufficient and thus no increase in biofilm formation could be observed. Overall, inhibition of peptidoglycan biosynthesis with subinhibitory concentrations of β-lactam antibiotics increased biofilm formation in distinct clinical NTHi isolates. These data are of particular importance because biofilm formation increases resistance to antibiotic treatment and clearance by the immune system (11), making these infections more difficult to treat.

In support of an important role for DNA in the extracellular matrix of *H. influenzae* biofilms, we show that DNase reduces biofilm formation by the R2866Δ*ampG* mutant, as well as WT R2866 treated with cefuroxime, confirming that extracellular DNA is required for the observed increase in biofilm formation. DNase is considered to be a useful adjunct treatment in children with recurrent or chronic OM (23), and our data support the detrimental effect on *H. influenzae* biofilms. Previously, we have shown that DNase treatment reduced *S. pneumoniae* bacterial loads in the middle ear in a mouse model (24). In fact, a clinical trial is determining whether DNase administered to the middle ears of children undergoing surgery for grommet insertion can resolve OM (25). Our data, in accordance with other *in vitro* studies (24, 26, 27), suggest that DNase treatment has potential as a therapeutic measure in the resolution of OM.

Altogether, we provide evidence that release of bacterial DNA through bacterial cell lysis contributes to biofilm formation *in vitro* and that interference with this could contribute to the clearance of NTHi biofilms.

## MATERIALS AND METHODS

### Bacterial strains and growth conditions.

The NTHi strains used in this study are listed in Table S1. The mutant library was generated by using NTHi strain 13/4, a biofilm-forming isolate from an oropharyngeal swab collected from a healthy child (18). Directed gene deletion mutants were generated in NTHi strain R2866 (28). NTHi was routinely grown with shaking at 37°C in brain heart infusion (BHI) broth (Becton Dickinson) supplemented with 10 µg/ml hemin (Sigma-Aldrich) and 2 µg/ml β-NAD (Merck) (sBHI). Growth on plates was performed with sBHI agar at 37°C and 5% CO_2_. Triton X-100 (Sigma) or cefuroxime (Calbiochem) was used to supplement sBHI at the concentrations mentioned in the figure legends.

10.1128/mSphere.00329-16.6 TABLE S1  Bacterial strains used in this study. Download TABLE S1, DOCX file, 0.1 MB. Copyright © 2017 Marti et al. 2017 Marti et al. This content is distributed under the terms of the Creative Commons Attribution 4.0 International license.

### Generation of the NTHi 13/4 transposon mutant library.

Genomic DNA was isolated from mid-log-phase cultures with the Qiagen DNeasy Blood and Tissue kit (Qiagen). The NTHi 13/4 *marinerT7*-MmeI transposon mutant library was generated as described previously for NTHi 86-028NP (29). The mutants were taken from sBHI plates and directly used in biofilm experiments.

### Static biofilm formation assay.

Biofilm formation was determined by static growth on 96-well plates and crystal violet staining as previously described (19). Proteinase K (100 μg/ml) or DNase (100 μg/ml) was added to sBHI at the start of the experiment.

To screen for biofilm formation by mutants, single colonies were diluted in 150 µl of sBHI and incubated at 37°C in 5% CO_2_ for 24 h. These experiments were performed with 1,806 and 2,366 colonies, and the data were combined, for a total of 4,172 colonies. In each tested plate, the biofilm formation and growth of 95 transposon mutants and the 13/4 parent strain were tested. Before biofilm staining, the OD_620_ was determined to assess bacterial growth and the culture supernatant was transferred to a new 96-well plate for mutant storage. Biofilm was stained as described above, and mutants that showed normal growth (defined as an OD_620_ <35% lower than that of the WT) but altered biofilm formation (defined as an *A*_560_ at least 1.5-fold different from that of the WT) were selected and stored at −80°C in 15% glycerol. The selected mutants were subjected to a second round of biofilm formation to corroborate phenotype differences. Because of the high number of false-positive transposon mutants in the first round, we performed a third round. In the third round, genomic DNA was isolated from each of the selected mutants with the Qiagen DNeasy Blood and Tissue kit (Qiagen) and crossed back into WT 13/4. At this point, the phenotypic modifications in these mutants were tested by performing a third round of biofilm formation.

### Identification of transposon insertions.

Transposon insertion was identified by single-primer PCR. One nanogram of genomic DNA was mixed with *Taq* PCR buffer, 2.5 mM MgCl_2_, 5 µl of a 250 µM deoxynucleoside triphosphate mixture, 20 µM primer (see Table S2), and 1 U of *Taq* DNA polymerase (total volume of 50 µl), and a PCR was run with the following program. Round 1 consisted of 30 s at 90°C, 30 s at 60°C, and 2 min at 72°C (30 cycles); round 2 consisted of 30 s at 90°C, 30 s at 30°C, and 2 min at 72°C (10 cycles); and round 3 consisted of 30 s at 90°C, 30 s at 55°C, and 2 min at 72°C (30 cycles), followed by 5 min at 72°C. The PCR product was purified with the Qiagen PCR purification kit (Qiagen). A 100-ng sample of the PCR product was mixed with 5 pmol of sequence primer (see Table S2)

10.1128/mSphere.00329-16.7 TABLE S2  Primers used in this study. Download TABLE S2, DOCX file, 0.1 MB. Copyright © 2017 Marti et al. 2017 Marti et al. This content is distributed under the terms of the Creative Commons Attribution 4.0 International license.

### Generation of NTHi directed gene deletion mutants.

Bacterial genomic DNA was isolated with the Qiagen DNeasy Blood and Tissue kit (Qiagen). Directed NTHi gene deletion mutants were generated by allelic exchange of the target gene with a spectinomycin resistance cassette as described previously (29). All of the primers (Biolegio) used in this study are listed in Table S2.

### Phase-contrast microscopy.

Bacteria grown in sBHI to an OD_620_ of 0.4 were centrifuged and washed twice with phosphate-buffered saline (PBS). The final bacterial pellet was completely resuspended in 100 µl of PBS to remove all bacterial clumps and fixed by adding 100 µl of 4% paraformaldehyde. The sample suspension was placed on a microscope slide and visualized with a Leitz Dialux 22 light microscope with a Nikon Coolpix camera.

### Confocal laser scanning microscopy.

Overnight bacterial cultures were used to inoculate an eight-well chambered cover glass for confocal image analysis (Ibidi GmbH) to a final OD_620_ of 0.01 in 2 ml of sBHI. The plates were incubated at 37°C for 24 h without shaking. After incubation, the wells were washed with distilled water and stained for 15 min in the dark with the cell-permeating fluorescent dye SYTO 17 Red Fluorescent Nucleic Acid Stain in accordance with the manufacturer’s instructions (Molecular Probes).

Samples were washed three times to remove nonspecific staining, and fluorescence was observed by confocal laser microscopy. Images of the double-labeled sections were acquired with a Leica TCS-SL filter-free spectral confocal laser scanning microscope (Leica Microsystems, Inc.) with a 488-nm argon laser, 543- and 633-nm He/Ne lasers (Centres Científics i Tecnològics, Campus de Bellvitge, Universitat de Barcelona, Barcelona, Spain), a 63× oil immersion objective (1.4 numerical aperture), and an image resolution of 1,024 by 1,024 pixels. The images were acquired randomly from the cover glass surface and analyzed with the Leica Confocal Software 2.5 (Leica Microsystems, Inc.).

### Sample preparation for AFM.

Biofilms were grown on Thermanox circular coverslips placed in 24-well microtiter plates covered with BHI medium and incubated at 37°C for different times. Afterward, the slides were washed three times in Milli-Q water and left to air dry for imaging.

### AFM imaging.

AFM measurements were performed in air with an XE-70 (Park Systems) at room temperature in noncontact mode with an ACTA silicon cantilever (Applied Nanostructures) with a nominal resonance frequency of 300 kHz and a nominal force constant of 37 N/m. Samples were grown as previously described and air dried for imaging. Measurements began by scanning a random area of 30 by 30 μm, which was gradually decreased until the bacterial surface could be observed in detail. Topography, amplitude, and phase images measuring 7.5 by 7.5 μm were recorded simultaneously. The acquired data were converted into topography, amplitude, and phase images and analyzed with XEI software (Park Systems). AFM imaging also allowed cell surface roughness measurement. A roughness average (Ra), meaning the average distance from the roughness profile to the center plane of the profile, was obtained from the acquired topography images.

### qPCR detection of genomic NTHi DNA.

Biofilms were scraped from the plate and suspended in 100 μl of PBS. One microliter of biofilm product was used in a qPCR. qPCR detection of the *Haemophilus* protein D gene (*hpd*) was performed with specific primers (see Table S2) in a 20-μl reaction volume with SYBR green PCR master mix (Bio-Rad) and a real-time PCR detection system (Bio-Rad).

### Bacterial adhesion assay.

The speed of initial bacterial adhesion was evaluated by the biofilm ring test on modified 96-well polystyrene plates obtained from BioFilm Control (St. Beauzire, France) as previously described (13). Briefly, bacterial suspensions were mixed with magnetic beads, incubated for 2 or 4 h at 37°C, and placed on a magnetic block. BioFilm Control software was used to obtain the biofilm formation index (BFI); values of >7 corresponded to a total lack of bacterial adherence, while values of <5 were associated with different degrees of bacterial adherence.

### A549 cell adhesion assay.

The human lung epithelial cell line A549 (ATCC CCL-185) was routinely grown in Dulbecco modified Eagle medium (DMEM) with GlutaMAX-I and 10% fetal calf serum (FCS; Invitrogen) at 37°C and 5% CO_2_. Two days prior to the adherence assay, 2 × 10^5^ A549 cells per well were seeded into a 24-well tissue culture plate, and after 1 day, the growth medium was refreshed. After 2 days of culturing, a confluent monolayer of approximately 1 × 10^6^ cells/well was formed. Bacteria were washed once in DMEM with GlutaMAX-I and 1% FCS (infection medium) and resuspended in the infection medium to 1 × 10^7^ CFU/ml. The A549 cells were washed twice with PBS and infected with 1 ml of the bacteria (multiplicity of infection, 10 bacteria per cell), which were allowed to adhere to the cells for 2 to 3 h at 37°C in a 5% CO_2_ environment. Nonadherent bacteria were removed by three washes with PBS, after which 1 ml of 1% saponin (Sigma-Aldrich) in PBS was added to detach and lyse the A549 cells. Bacterial counts were determined at the start (inoculum), after 2 to 3 h of adhesion (total number of bacteria), and after lysis in saponin (number of bacteria that adhered to and invaded cells). The percentage of bacteria that adhered to and invaded cells was calculated by dividing the number of bacteria that adhered to and invaded cells by the total number of bacteria plus the number of bacteria that adhered to and invaded cells.

### Aggregation assay.

NTHi was grown shaking at 37°C in sBHI to an OD_620_ of ~0.9. Subsequently, 2 ml of culture was incubated without shaking at 37°C in 5-ml tubes. To determine sedimentation as a result of aggregation, the OD_620_ of the upper part of the tube was determined at 0, 2, 4, and 8 h.

### Statistical analyses.

Statistical analyses were performed with GraphPad Prism version 5.03 for Windows (GraphPad Software, Inc.). Differences were considered significant at *P* < 0.05. The specific statistical tests that were used for the various experiments are specified in the figure legends.
