# Supplementary figures and images for: Bacterial Lysis through Interference with Peptidoglycan Synthesis Increases Biofilm Formation by Nontypeable Haemophilus influenzae
Source: mSphere. 2017 Jan 18;2(1):e00329-16. doi: 10.1128/mSphere.00329-16 (PMC5244263; doi:10.1128/mSphere.00329-16)

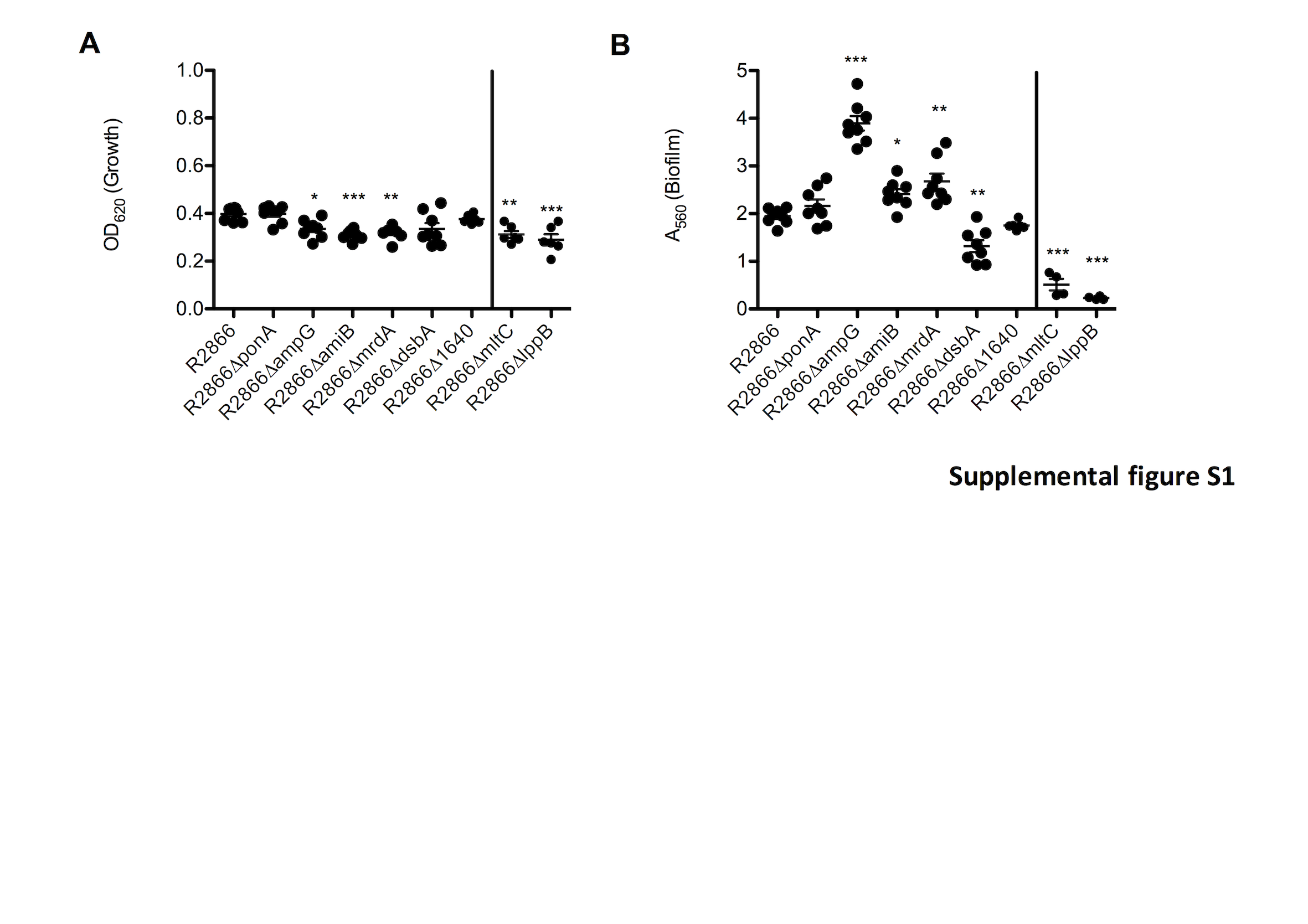

Supplement: FIG S1 [file sph001172225sf3.tif]

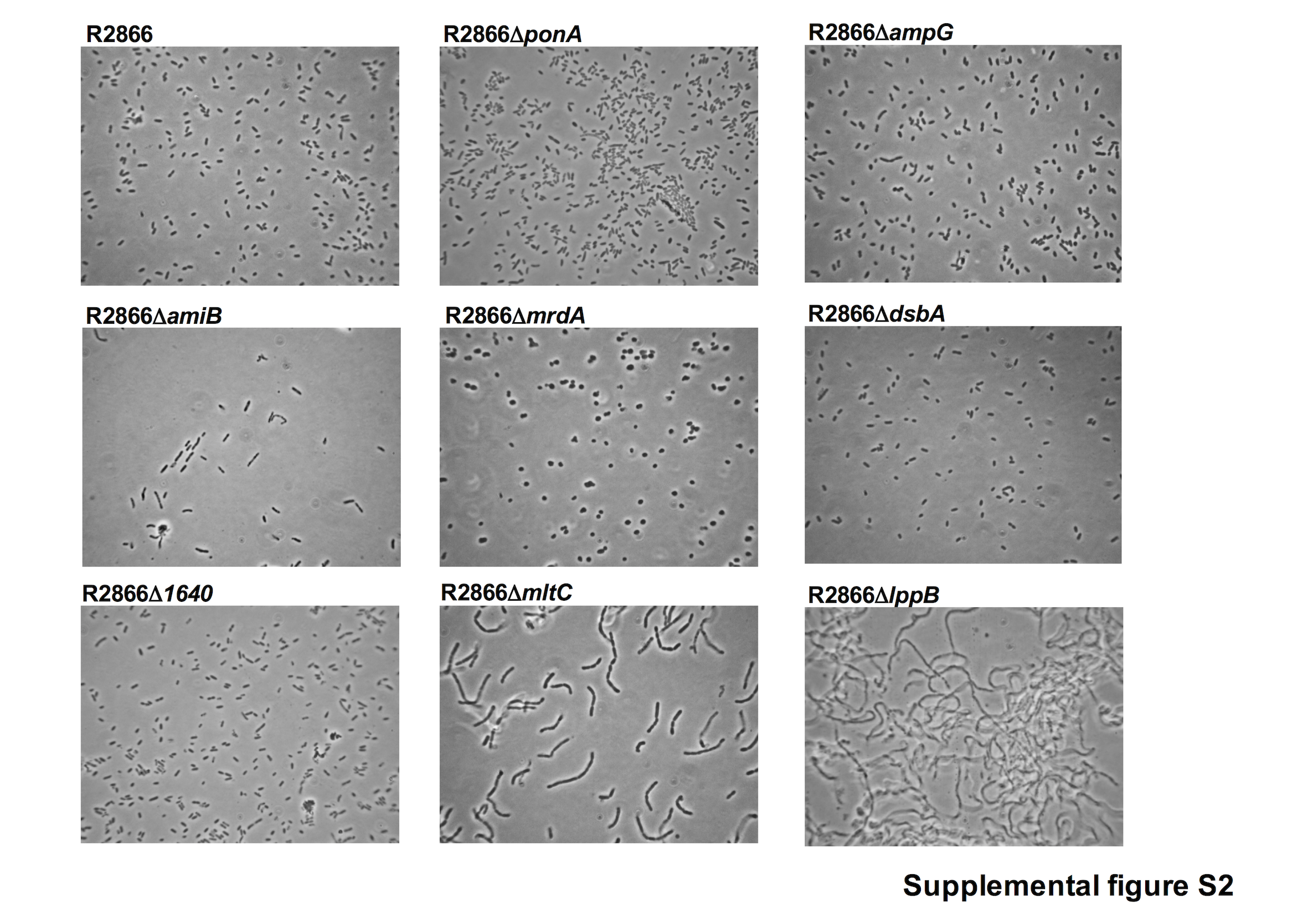

Supplement: FIG S2 [file sph001172225sf4.tif]

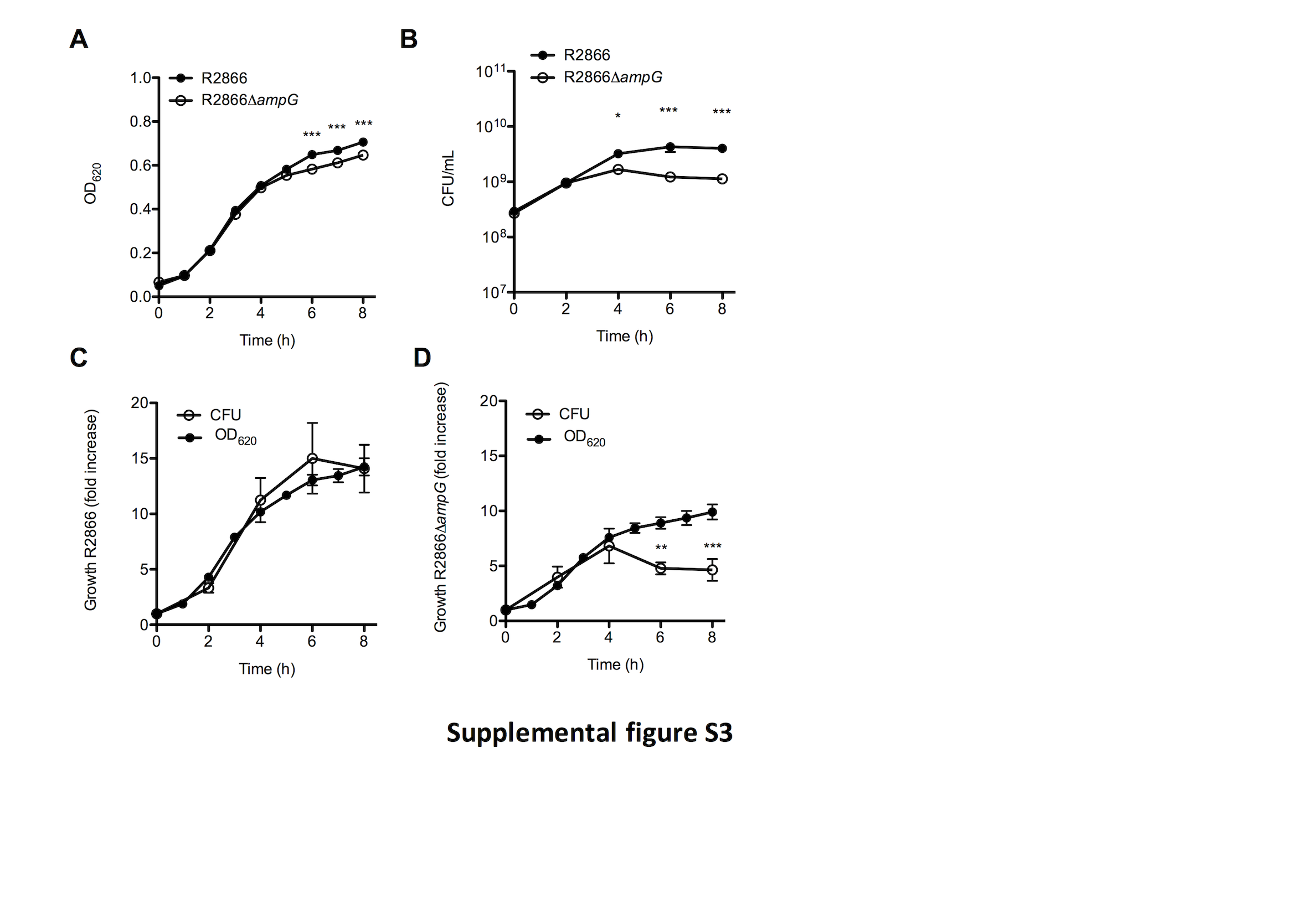

Supplement: FIG S3 [file sph001172225sf5.tif]

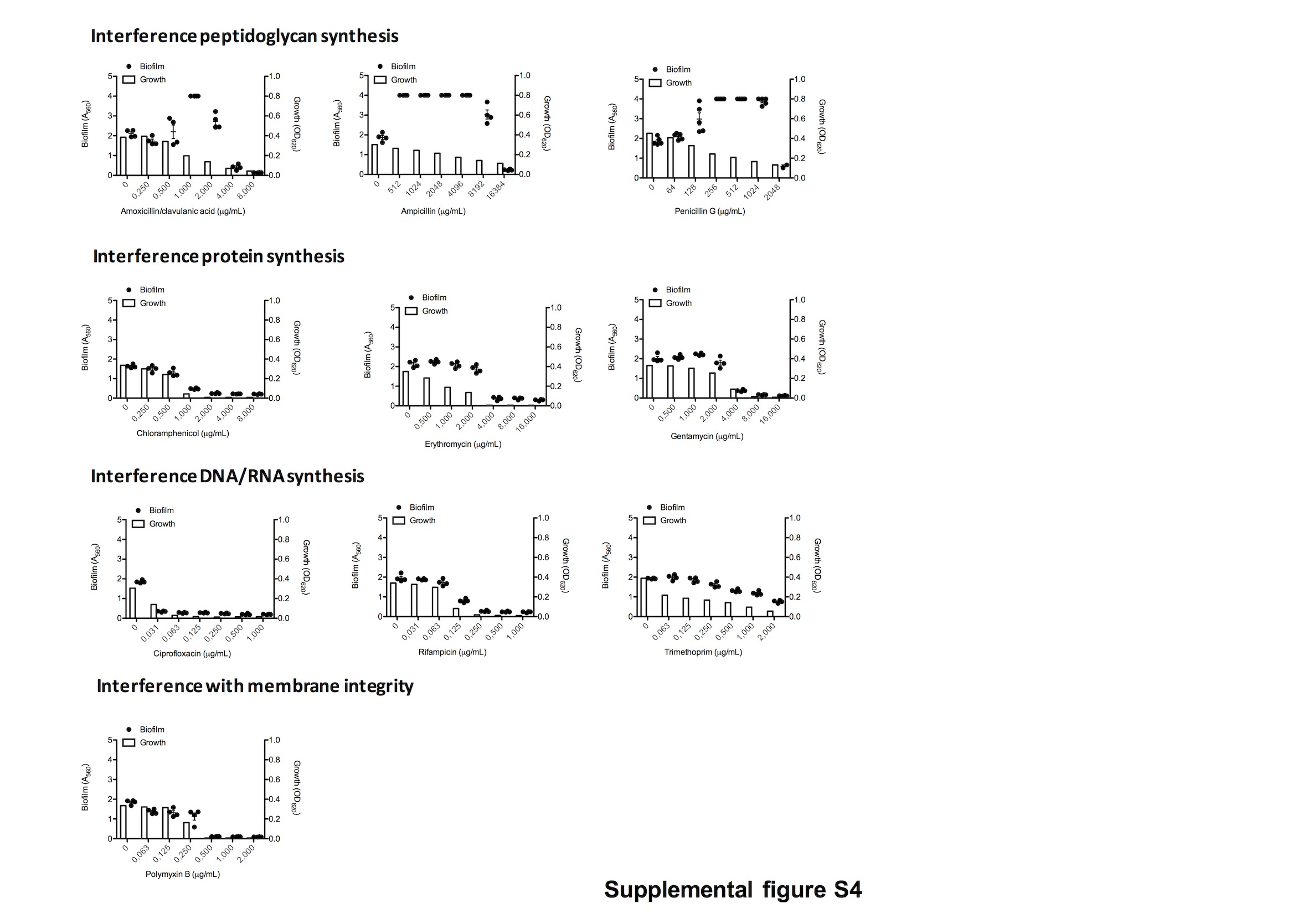

Supplement: FIG S4 [file sph001172225sf6.tif]

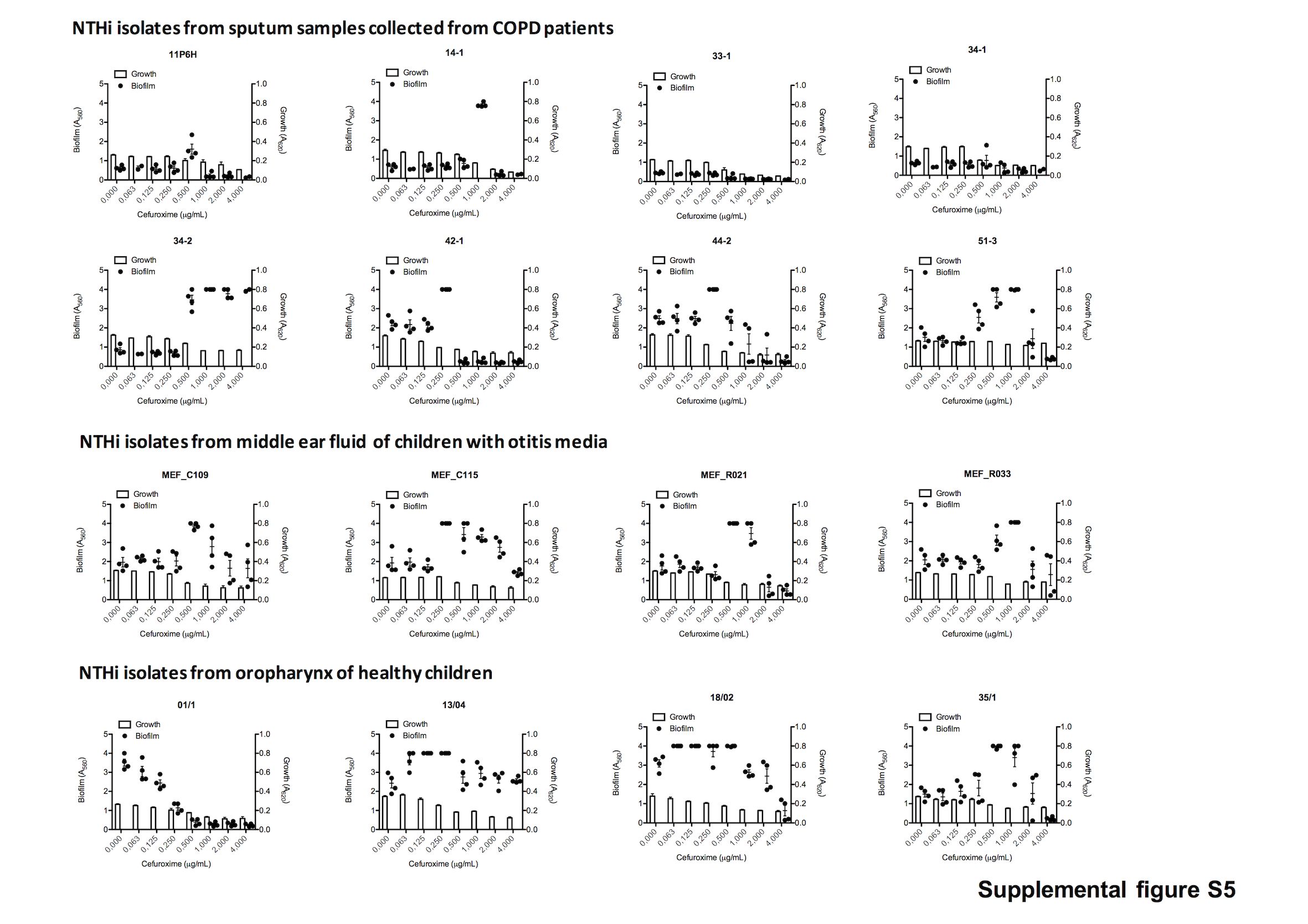

Supplement: FIG S5 [file sph001172225sf7.tif]
