# Supplementary material for: Bacterial Lysis through Interference with Peptidoglycan Synthesis Increases Biofilm Formation by Nontypeable Haemophilus influenzae
Source: mSphere. 2017 Jan 18;2(1):e00329-16. doi: 10.1128/mSphere.00329-16 (PMC5244263; doi:10.1128/mSphere.00329-16)
Supplement: TABLE S1 [file sph001172225st1.docx]

| **Supplemental Table S1.** Bacterial strains used in this study. | | |
| --- | --- | --- |
|  |  |  |
| **Strain name** | **Description** | **Reference** |
| 01/1, 13/04, 18/02, 35/1 | NTHi isolates from oropharynx of healthy child in day care center | C. Puig et al., Microb. Drug Resist. 2014 Oct;20(5):450-5. |
| 14-1, 33-1, 34-1, 34-2, 42-1, 44-2, 51-3 | NTHi isolates from sputum samples collected from COPD patients | C. Puig et al., Infect. Immun. 2014 Apr;82(4):1591-9. |
| MEF_C109, MEF_C115, MEF_R021, MEF_R033 | NTHi isolates from middle ear fluid of children with otitis media | J.D. Langereis et al., Med Microbiol. Immunol. 2013 Dec;202(6):407-15 |
| 11P6H | NTHi clinical isolate from sputum of an adult COPD patient during an acute exacerbation | K. Yi et al., J Infect Dis. 1997 Nov;176(5):1247-52. |
| R2866 | Invasive NTHi clinical isolate collected from the blood | B.J. Williams et al., Infec. Immun. 2001 Feb;69(2):695-705. |
| R2866Δ*dsbA* | R2866 with *dsbA* gene replaced by Spec^r^ cassette | This study |
| R2866Δ*ampG* | R2866 with ampG gene replaced by Spec^r^ cassette | This study |
| R2866Δ*mrdA* | R2866 with mrdA gene replaced by Spec^r^ cassette | This study |
| R2866Δ*ponA* | R2866 with ponA gene replaced by Spec^r^ cassette | This study |
| R2866Δ*amiB* | R2866 with amiB gene replaced by Spec^r^ cassette | This study |
| R2866Δ*1640* | R2866 with *R2866_1640* gene replaced by Spec^r^ cassette | This study |
| R2866Δ*mltC* | R2866 with *mltC* gene replaced by Spec^r^ cassette | This study |
| R2866Δ*lppB* | R2866 with *lppB* gene replaced by Spec^r^ cassette | This study |
