# Supplementary material for: Bacterial Lysis through Interference with Peptidoglycan Synthesis Increases Biofilm Formation by Nontypeable Haemophilus influenzae
Source: mSphere. 2017 Jan 18;2(1):e00329-16. doi: 10.1128/mSphere.00329-16 (PMC5244263; doi:10.1128/mSphere.00329-16)
Supplement: TABLE S2 [file sph001172225st2.docx]

| **Supplemental Table S2.** Primers used in this study. | | |  |
| --- | --- | --- | --- |
|  |  |  |  |
| **Primer name** | **Description** | **Sequence** | **Reference** |
| PBpR412_L | For specr amplification | GATACCCCTCGAATTGACGC | P. Burghout *et al*., J Bacteriol. 2007 Sep;189(18):6540-50. |
| PBpR412_R | For specr amplification | CAATGGTTCAGATACGACGAC | P. Burghout *et al*., J Bacteriol. 2007 Sep;189(18):6540-50. |
| PBMrTn9 | For gene replacement control PCR | CAATGGTTCAGATACGACGAC | S. P. de Vries *et al*., Mol Microbiol. 2013 Jan;87(1):14-29. |
| R2866_0135_L1 | For megaprimer PCR *ponA* gene replacement | CCACCCACTTTTTGTTGATG | This study |
| R2866_0135_L2 | For megaprimer PCR *ponA* gene replacement | CCACTAGTTCTAGAGCGGCAGCATTCCGCCTGCTACTAA | This study |
| R2866_0135_R1 | For megaprimer PCR *ponA* gene replacement | CGGTGAAATTTAGTGGCGTTT | This study |
| R2866_0135_R2 | For megaprimer PCR *ponA* gene replacement | GCGTCAATTCGAGGGGTATCCGACGCAACCACAAGAACT | This study |
| R2866_0135_C | For ponA gene replacement control PCR | CTCACCCACTTCGCCAATTA | This study |
| R2866_0223_L1 | For megaprimer PCR *ampG* gene replacement | TGCAGGGTTAAAAGCCGTAG | This study |
| R2866_0223_L2 | For megaprimer PCR *ampG* gene replacement | CCACTAGTTCTAGAGCGGCCTTGCGCGTAAAAATTTGAG | This study |
| R2866_0223_R1 | For megaprimer PCR *ampG* gene replacement | CCTGATATGAGTGGCGTTGA | This study |
| R2866_0223_R2 | For megaprimer PCR *ampG* gene replacement | GCGTCAATTCGAGGGGTATCGATTCCTGGCATGCTTTGTT | This study |
| R2866_0223_C | For *ampG* gene replacement control PCR | CATCACACCCGTTACTGCAC | This study |
| R2866_0638_L1 | For megaprimer PCR amiB gene replacement | TCCACAAGATCAACCCGATT | This study |
| R2866_0638_L2 | For megaprimer PCR amiB gene replacement | CCACTAGTTCTAGAGCGGCTGGAGCTGCAAAAAGAGAAA | This study |
| R2866_0638_R1 | For megaprimer PCR amiB gene replacement | AGGCTTGTCGAATAGCATGG | This study |
| R2866_0638_R2 | For megaprimer PCR amiB gene replacement | GCGTCAATTCGAGGGGTATCTGGCAAAGTTATTACAGGACAAA | This study |
| R2866_0638_C | For amiB gene replacement control PCR | CCTAAATTTCGGCTGATTGC | This study |
| R2866_0673_L1 | For megaprimer PCR mrdA gene replacement | TTTTGCGCAAGAGGGTATTC | This study |
| R2866_0673_L2 | For megaprimer PCR mrdA gene replacement | CCACTAGTTCTAGAGCGGCGTTCGTGCGTTGGTGTATTG | This study |
| R2866_0673_R1 | For megaprimer PCR mrdA gene replacement | AGCAAGAATACGCCCAAAAG | This study |
| R2866_0673_R2 | For megaprimer PCR mrdA gene replacement | GCGTCAATTCGAGGGGTATCTTTGTCACAAGAGAGCGAACA | This study |
| R2866_0673_C | For mrdA gene replacement control PCR | TTACCATTAGAACGCGTTTGG | This study |
| R2866_1548_L1 | For megaprimer PCR dsbA gene replacement | TGCGATATTTGGTGCTCGTA | This study |
| R2866_1548_L2 | For megaprimer PCR dsbA gene replacement | CCACTAGTTCTAGAGCGGCGCGTGCTGACACCTAAACCT | This study |
| R2866_1548_R1 | For megaprimer PCR dsbA gene replacement | CACGCCCGATTATTTGAAC | This study |
| R2866_1548_R2 | For megaprimer PCR dsbA gene replacement | GCGTCAATTCGAGGGGTATCCGTGAAAGATTATGTGCAAACC | This study |
| R2866_1548_C | For dsbA gene replacement control PCR | TTTTGCTGTGAAGCCTGTTG | This study |
| R2866_1640_L1 | For megaprimer PCR R2866_1640 gene replacement | ACAGCCGTTTCAAAATTCCA | This study |
| R2866_1640_L2 | For megaprimer PCR R2866_1640 gene replacement | CCACTAGTTCTAGAGCGGCTAACGCCAAAACGCAACATA | This study |
| R2866_1640_R1 | For megaprimer PCR R2866_1640 gene replacement | GTGGACAAAGCAGTTGTTAATG | This study |
| R2866_1640_R2 | For megaprimer PCR R2866_1640 gene replacement | GCGTCAATTCGAGGGGTATCGGGGAAATATCACGTTCTGC | This study |
| R2866_1640_C | For R2866_1640 gene replacement control PCR | GAGATCGGAAGATTGGCTGA | This study |
| R2866_1635_L1 | For megaprimer PCR mltC gene replacement | TGGTAATGTGAAACGTGTGTTGG | This study |
| R2866_1635_L2 | For megaprimer PCR mltC gene replacement | CCACTAGTTCTAGAGCGGCAACCCTTGCGTGTCTTTAGC | This study |
| R2866_1635_R1 | For megaprimer PCR mltC gene replacement | GCCAATATAGCCCACAGGAG | This study |
| R2866_1635_R2 | For megaprimer PCR mltC gene replacement | GCGTCAATTCGAGGGGTATCCGACAGTTCACCCATCATCA | This study |
| R2866_1635_C | For mltC gene replacement control PCR | TTTGGATCTTGCTGGGTTTC | This study |
| R2866_1770_L1 | For megaprimer PCR lppB gene replacement | TTATTGGATTGGCCCTTCAG | This study |
| R2866_1770_L2 | For megaprimer PCR lppB gene replacement | CCACTAGTTCTAGAGCGGCTCAAAGGGAGTAAGAGAAACGA | This study |
| R2866_1770_R1 | For megaprimer PCR lppB gene replacement | ACCACCCGCAGAGTTACAAG | This study |
| R2866_1770_R2 | For megaprimer PCR lppB gene replacement | GCGTCAATTCGAGGGGTATCTCAGTGGATCCAGTACGTTACC | This study |
| R2866_1770_C | For lppB gene replacement control PCR | TGCCAAGCACCACCTACAT | This study |
| hpdF729 | For NTHi genomic DNA quantification | AGATTGGAAAGAAACACAAGAAAAAGA | X. Wang et al., Int J Med Microbiol. 2011 Apr;301(4):303-9. |
| hpdR819 | For NTHi genomic DNA quantification | CACCATCGGCATATTTAACCACT | X. Wang et al., Int J Med Microbiol. 2011 Apr;301(4):303-9. |
| PBMrTn8 | Primer for single primer PCR | CGGGAATCATTTGAAGGTTGG | P. Burghout et al., J Bacteriol. 2013 Apr;195(7):1573-82. |
| PBMrTn1 | Sequence primer for single primer PCR product | CTAGCGACGCCATCTATGTG | T.G. Kloosterman et al., J Bacteriol. 2008 Aug;190(15):5382-93. |
